# A Case of Epilepsy, for Which the Patient Was Trephined

**Published:** 1856-04

**Authors:** D. Hayes Agnew

**Affiliations:** Surgeon to the Philadelphia Hospital, Blockley; Philadelphia


*A case of Epilepsy, for which the patient was Trephined.* By D. Hayes Agnew, M. D., Surgeon to the Philadelphia Hospital, Blockley.

Just conclusions in medicine can only be reached by a faithful record of facts; and in no case is this more necessary than where, from the circumstances attending the disease, the patient is likely to be subjected to the hazards of an operation.

It is on this account I place on record the following case: George Gerlach, a German, 24 years of age, of sanguine temperament, and remarkable physical developement, was admitted into the lunatic department of the Philadelphia Hospital, laboring under a mild form of insanity. Eleven years previous to his admission, and before coming to this country, he received a violent stroke upon the top of the head by a falling piece of timber. As far as can be gleaned from his friends, no symptoms of compression existed at the time; but two or three months subsequently, he was attacked with epileptic convulsions, which becoming more and more frequent and violent, his mind at length began to suffer materially as the result. During my term of service in the Institution, he came under my notice. His attacks were very violent, and as frequent as two or three every week. Examining his head I discovered, close along the position and direction of the sagittal suture, a well marked depression one inch and a half in length, and in which the little finger could be laid. At my suggestion his friends readily agreed there should be something attempted for his relief. Having placed him under the influence of ether, he was brought before the class in attendance, the parts shaven, the bone well exposed and by a large sized trephine the most depressed portion of the bone resected. The dura mater was very firmly attached to the piece, but by careful manipulation was separated without the least injury to the membrane. During the operation he was seized with a very violent convulsion, which lasted for several minutes. The external table of the part removed, presented two considerable depressions. The internal table exhibited the fissures of a beautiful stellated fracture, while both, together with the diploe corresponding to the two indentations, had almost disappeared, existing only as a thin lamina. If any undue pressure did exist, of which I have no doubt, it was the result of the absorption of the parts referred to, rendering the other portion more prominent. The parts having been dressed, the man was placed in the clinical ward ; a water dressing to the wound and small doses of hydrarg. chlor. mit. prescribed, with a view to counteract the dangers of meningeal inflammation. He recovered without a single untoward symptom. The first week succeeding the operation, he had two convulsions, after which they did not recur more than once a week, and sometimes once in two weeks, and then very mild compared with those previous to the resection. Being very much relieved in all respects, he left the hospital, and, as far as I can learn at the present time, continues materially benefited. Time can only test the permanency of the relief. If the piece of bone removed was the primary cause of the epilepsy, we would have no reason to argue an unfavorable prognosis because the attacks did not entirely subside, as the long continuance of that exciting cause would have a tendency to develope a condition of the encephalic mass, calculated to continue for a time at least from habit.

*Philadelphia, March 12th*, 1856.

